# Lower health literacy predicts smoking relapse among racially/ethnically diverse smokers with low socioeconomic status

**DOI:** 10.1186/1471-2458-14-716

**Published:** 2014-07-14

**Authors:** Diana W Stewart, Miguel Ángel Cano, Virmarie Correa-Fernández, Claire Adams Spears, Yisheng Li, Andrew J Waters, David W Wetter, Jennifer Irvin Vidrine

**Affiliations:** 1 The Department of Health Disparities Research, The University of Texas MD Anderson Cancer Center, Houston, TX, USA; 2 The Department of Psychology, The Catholic University of America, Washington, DC, USA; 3 The Department of Biostatistics, The University of Texas MD Anderson Cancer Center, Houston, TX, USA; 4 The Department of Medical and Clinical Psychology, Uniformed Services University of the Health Sciences, Bethesda, MD, USA; 5 The Department of Psychology, Rice University, Houston, TX, USA

**Keywords:** Health literacy, Smoking cessation, Health disparities

## Abstract

**Background:**

Nearly half of U.S. adults have difficulties with health literacy (HL), which is defined as the ability to adequately obtain, process, and understand basic health information. Lower HL is associated with negative health behaviors and poor health outcomes. Racial/ethnic minorities and those with low socioeconomic status (SES) are disproportionately affected by poor HL. They also have higher smoking prevalence and more difficulty quitting smoking. Thus, lower HL may be uniquely associated with poorer cessation outcomes in this population.

**Methods:**

This study investigated the association between HL and smoking cessation outcomes among 200, low-SES, racially/ethnically diverse smokers enrolled in smoking cessation treatment. Logistic regression analyses adjusted for demographics (i.e., age, gender, race/ethnicity, relationship status), SES-related characteristics (i.e., education, income), and nicotine dependence were conducted to investigate associations between HL and smoking relapse at the end of treatment (3 weeks post quit day).

**Results:**

Results indicated that smokers with lower HL (score of < 64.5 on the Rapid Estimate of Adult Literacy in Medicine [REALM]) were significantly more likely than those with higher HL (score of ≥ 64.5 on the REALM) to relapse by the end of treatment, even after controlling for established predictors of cessation including demographics, SES, and nicotine dependence (OR = 3.26; 95% CI = 1.14, 9.26).

**Conclusions:**

Findings suggest that lower HL may serve as an independent risk factor for smoking relapse among low-SES, racially/ethnically diverse smokers enrolled in treatment. Future research is needed to investigate longitudinal relations between HL and cessation outcomes and potential mechanisms of this relationship.

## Background

For over 90 million U.S. adults, difficulty with health literacy (HL) interferes with the capacity to obtain, process, and understand basic health information needed to make appropriate healthcare decisions [1,2]. Thus, HL is a conceptually-distinct construct from poor knowledge regarding smoking-related health risks. Individuals with lower HL are more likely to engage in unhealthy behaviors (e.g., unhealthy eating, poor medication adherence), and they have less access to prevention and treatment programs [3,4]. Compared to individuals with higher HL, those with lower HL are more likely to have poor health status and health outcomes [4]. They are also more likely to be of low socioeconomic status (SES) and racial/ethnic minorities [5]. Because smoking is the leading behavioral risk factor contributing to social disparities in the incidence and mortality of disease [6-10], there is a critical need to understand how difficulty with HL might impact smoking cessation outcomes. It is possible that HL is an important, but overlooked, factor in understanding tobacco-related health disparities.

To date, little is known about the potential impact of low HL on smoking and cessation outcomes. Sudore and colleagues reported a significant relationship between lower HL and current smoking status among elderly adults [11]; however, Baker et al. found no association between lower HL and smoking status in a different sample of older adults [12]. Arnold and colleagues reported no association between HL and smoking status among low-income, pregnant women, yet found that lower HL was significantly associated with lower levels of smoking risk knowledge and fewer negative smoking-related attitudes [13]. A more recent study found that among low-SES, racially/ethnically diverse smokers, lower HL was significantly associated with certain established predictors of smoking relapse (i.e., nicotine dependence, stronger positive and weaker negative smoking outcome expectancies, less smoking health risk knowledge, and lower smoking risk perceptions), even after controlling for demographic and SES-related characteristics known to predict relapse i.e., age, gender, race/ethnicity, education, income, relationship status; [14].

These findings highlight the need for studies to investigate the association between HL and cessation outcomes, particularly among racially/ethnically diverse smokers. Smokers with lower HL are more difficult to recruit and retain in treatment studies e.g., [15]. Furthermore, smokers with lower HL who do enroll in treatment may have trouble understanding oral and written treatment materials, and may fail to tell others about these difficulties due to shame and embarrassment see [16,17]. Research is needed to gain a better understanding of the association between HL and smoking cessation outcomes and potential mechanisms of this association. Such studies would help to inform and improve smoking prevention and cessation interventions targeting individuals with HL difficulties, and should ultimately contribute to reducing tobacco-related health disparities.

The purpose of the present study was to examine the impact of HL on smoking cessation outcomes among low-SES, racially/ethnically diverse smokers enrolled in cessation treatment. Based on prior research, it was hypothesized that lower HL would be associated with poorer cessation outcomes, even after controlling for demographic (i.e., age, gender, race/ethnicity, relationship status) and SES-related characteristics (i.e., education, income) and nicotine dependence.

## Method

### Participants and eligibility criteria

Participants (N = 200) were daily smokers enrolled in a larger smoking cessation treatment study (Project PRISM) designed to investigate changes in smoking risk perceptions throughout the process of quitting smoking. The main outcome paper for this trial is currently in preparation. All participants were recruited in Houston, TX via media (e.g., paid advertisements, public service announcements) and community outreach (e.g., staff visits to healthcare setting and health fairs, distribution of study flyers). Eligible participants smoked ≥ 5 cigarettes per day for the last year, had expired carbon monoxide (CO) levels of ≥ 8 parts per million (ppm), were able to read at ≥ 6th grade reading level, and were between 18–65 years old. Exclusion criteria were: contraindication for nicotine patch use, current diagnosis of substance abuse or dependence, use of nicotine replacement therapy or bupropion products other than the nicotine patches provided by the study, regular use of tobacco products other than cigarettes, enrollment of other household members in the study, and pregnancy or lactation.

### Procedure

Study staff contacted interested smokers by phone. After receiving a detailed description of the study, potential participants provided verbal informed consent and were screened for eligibility. Eligible participants were scheduled for in-person baseline orientation visits during which the study was further described, written consent was obtained, eligibility was finalized, and baseline measures were completed. All questionnaires were administered in private interview rooms via the Questionnaire Design System (QDS), a computer-administered self-report interview system that includes audio and visual scripts (i.e., participants are able to hear and read questions).

Participants attended five in-person study visits (i.e., baseline, week 0 [quit day], weeks 1–3 post-quit). Smoking cessation treatment for all participants comprised four weeks of nicotine patch therapy, four brief in-person counseling sessions based on the *Treating Tobacco Use and Dependence Clinical Practice Guideline*[18], and self-help materials. This study was approved by the Institutional Review Board at the University of Texas MD Anderson Cancer Center.

### Measures

Demographics and indicators of socioeconomic status were assessed at baseline and included age, gender, race/ethnicity, education, total annual household income, and relationship status. Responses for the following variables were categorized as: gender (male, female), race/ethnicity (Non-Latino White, Black, Other), education (< high school degree vs. ≥ high school degree/GED), income (< $20,000 vs. ≥ $20,000), and relationship status (married/living with a partner vs. not married/not living with a partner). Age was treated continuously.

Health literacy was measured at baseline with the Rapid Estimate of Adult Literacy in Medicine (REALM), a word recognition test that that assesses whether individuals have the ability to read and correctly pronounce 66 common medical words and lay terms for body parts [19]. Individuals are instructed to read through the list of words and to pronounce as many words as possible. Scoring is based on standard dictionary pronunciation rules. The sum of words read correctly is translated into one of four grade level estimates (0–18, < 4^th^ grade; 19–44, 4^th^-6^th^ grade; 45–60, 7^th^-8^th^ grade; ≥ 61, ≥ 9^th^ grade). The REALM is one of the most commonly used measures of HL [20]. It has excellent test-retest reliability and is highly correlated with comprehensive literacy measures such as the Wide Range Achievement Test-Revised and tests of functional HL such as the Test of Functional Health Literacy in Adults [19,21,22].

Nicotine dependence was assessed at baseline via the heaviness of smoking index (HSI), which is comprised of two items: self-reported average number of cigarettes smoked per day and time to first cigarette upon waking [23]. The HSI is a good indicator of nicotine dependence, has fair internal consistency [24], and predicts smoking relapse [25].

Biochemically-verified (CO < 8 ppm), self-reported continuous smoking abstinence was assessed at the end of treatment (3 weeks post quit). Continuous abstinence was defined as no smoking, not even a puff, since the quit date. Self-reported abstinence was assessed one week, two weeks, and three weeks following the quit day, and was biochemically verified at each of the three assessment points using expired air carbon monoxide levels (CO < 8 ppm). Participants who reported abstinence from smoking at any of these assessments but had expired air CO levels > 8 ppm were classified as smoking. The Society for Nicotine and Tobacco Subcommittee on Biochemical Verification [26] has indicated that a CO reading of 8–10 ppm distinguishes nonsmokers from smokers.

### Statistical analyses

Bivariate correlations examined associations among HL, demographics, nicotine dependence, and cessation outcomes. Logistic regression analyses adjusted for demographics (i.e., age, gender, race/ethnicity, relationship status) and SES (i.e., education, income) and nicotine dependence were used to examine associations between HL and smoking relapse.

## Results

Participants had a mean age of 46.1 years (*SD* = 9.7) and were primarily female and non-Latino White (see Table 1). REALM scores were quite high (mean = 62.3; SD = 5.4; range = 41 – 66). Therefore, consistent with multiple previous studies, we dichotomized HL [14,27-29] based on a median split (lower HL < 64.5 vs. higher HL ≥ 64.5). Lower HL was associated with being Black and having lower education and income (*p*s < .05). See Table 2 for correlations among study variables.

**Table 1:** Participant characteristics

**Variable**	**Total sample (N = 200), Mean (SD) or No. (%)**
Age (years; range 18–69)	46.1 (9.7)
Gender	
Female	115 (57.5)
Male	85 (42.5)
Race	
Non-Latino White	102 (51.0)
Black	89 (44.5)
Other	9 (4.5)
Total annual household income	
< $20,000	89 (44.5)
≥ $20,000	106 (53.0)
Missing	5 (2.5)
Educational level	
< High school degree/GED	64 (32.0)
≥ High school degree/GED	136 (68.0)
Relationship status	
Married/living with partner	58 (29.0)
Not married/not living with partner	142 (71.0)
REALM	62.3 (5.4)
HSI	3.7 (1.3)
Cessation outcome	
Relapse	170 (85.0)
Abstinent	30 (15.0)

*Note.* REALM = Rapid Estimate of Adult Literacy in Medicine. HSI = Heaviness of Smoking Index.

**Table 2:** Correlations among study variables

**Variables**	**REALM**	**Age**	**Gender**	**Race**	**Income**	**Education**	**Relationship status**	**HSI**	**Smoking relapse**
REALM	-								
Age	-.09	-							
Gender^a^	.11	-.13	-						
Race^b^	-.32**	-.02	-.05	-					
Income^c^	.22**	.08	-.17*	-.21**	-				
Education^d^	.28**	.12	-.13	-.14*	.25**	-			
Relationship Status^e^	.02	.01	-.34**	-.10	.38**	.13	-		
HSI	-.10	.20**	.03	-.15*	-.04	-.22**	.06	-	
Smoking Relapse^f^	-.18*	-.01	.14	.14	-.18*	-.14	-.22**	.06	-

*Note.* REALM = Rapid Estimate of Adult Literacy in Medicine. HSI = Heaviness of Smoking Index. ^a^Gender: 0 = male, 1 = female; ^b^Race: 0 = non-Latino White, 1 = Black/Other racial/ethnic minority; ^c^Total Annual Household Income: 0 = < $20,000, 1 ≥ $20,000; ^d^Education: 0 = < high school degree/GED, 1 ≥ high school degree/GED; ^e^Relationship Status: 0 = not married/not living with partner; 1 = married/living with partner; ^f^Smoking Relapse: 0 = abstinent, 1 = relapsed.

*p < .05; **p < .01.

Logistic regression analyses adjusted for demographics, SES, and nicotine dependence indicated that smokers with lower (vs. higher) HL were significantly more likely to relapse by the end of treatment (OR = 3.26; 95% CI = 1.14, 9.26; See Table 3).

**Table 3:** Logistic regression analyses of the association between health literacy and smoking relapse, controlling for age, gender, race/ethnicity, income, education, relationship status, and nicotine dependence

**Variable**	**Odds ratio (OR)**	**95% Confidence interval (CI)**
Health Literacy	3.26	1.15 – 9.26*
Age	.98	.93 – 1.23
Gender		
Female	.46	.17 – 1.23
Male	2.13	.79 – 5.71
Race/Ethnicity		
Black	.52	.20 – 1.37
Non-Latino White	1.93	.73 – 5.13
Income		
<$20,000	.84	.28 – 2.53
≥$20,000	1.16	.39 – 3.45
Education		
< High school degree/GED	.65	.19 – 2.23
≥ High school degree/GED	1.49	.43 – 5.16
Relationship Status		
Married/living with partner	2.21	.78 – 6.23
Not married/not living with partner	.47	.17 – 1.34
Nicotine Dependence	1.24	.86 – 1.79

*Note.* The odds ratios are adjusted for all demographic covariates (i.e., age, gender, race/ethnicity, income, education, relationship status, nicotine dependence).

*p < .05.

## Discussion

To our knowledge, this is the first known investigation of the association between HL and smoking cessation outcomes among low-SES, racially/ethnically diverse smokers enrolled in cessation treatment. As hypothesized, smokers with lower HL were more likely than smokers with higher HL to relapse following cessation treatment. Importantly, this association held after adjusting for established predictors of relapse including nicotine dependence, demographics, and SES (i.e., education, income). Findings provide preliminary support for lower HL as an independent risk factor for smoking relapse among low-SES, racially/ethnically diverse smokers.

Results indicated that lower HL was associated with being Black and having lower education and income. These findings are consistent with the 2003 National Assessment of Adult Literacy [1,30], which indicated that lower HL was associated with male gender, older age, racial/ethnic minority status, and SES-related characteristics such as lower education and income. They are also in line with results from numerous other studies [11,14,31-33]. It is noteworthy that lower (vs. higher) HL smokers in this study were more than three times as likely to relapse, even after controlling for the effects of established predictors of relapse. These results support prior research suggesting that HL is a crucial SES-related factor essential in understanding health disparities [4,14,32,34].

In addition to being associated with a greater likelihood of smoking relapse, prior research has found that lower HL is associated with higher nicotine dependence, more positive and fewer negative expectancies about the consequences of smoking, less knowledge about smoking health risks, and lower smoking risk perceptions [13,14]. These findings highlight the need for efforts to increase awareness about the impact of HL difficulties on smoking, and potentially other, poor health behaviors. Healthcare providers should be trained to communicate clearly with patients about the health consequences of smoking, and to offer treatments that do not require high HL. Regardless of HL level, providers should utilize plain language, visual aids (e.g., pictographs), and techniques such as the teach-back method to convey smoking health risks [33,35]. Notably, research is needed to investigate potential mechanisms underlying the association between HL and cessation outcomes among low-SES, racially ethnically diverse smokers. Such findings could be useful in the development of cessation interventions targeting smokers with HL difficulties, and thereby help to reduce tobacco-related health disparities.

The present study has several limitations. First, the larger study required that eligible participants have at least a sixth grade reading level. This eligibility criterion resulted in a range of scores on the REALM that was skewed toward the higher end of the HL continuum, and individuals with the lowest levels of health literacy were not well represented in the study. However, it is notable that this eligibility criterion resulted in the exclusion of only six individuals screened. This phenomenon is consistent with prior research suggesting that smokers with lower HL might be more difficult to recruit and retain in treatment studies e.g., [15]. Due to the restricted range of the REALM, we were unable to use the traditional cut-off points, and dichotomized the REALM based on a median split. Notably, despite this restricted range, a significant association between HL and smoking relapse emerged. Given this restricted range, it is possible that the association between HL and smoking relapse may have been underestimated, and future studies are needed to examine this association among smokers with levels of HL that are lower than those observed in the current study. Nevertheless, it is important to note that the 95% confidence interval was relatively wide (1.15 – 9.26), suggesting that this finding should be interpreted cautiously.

An additional limitation is that long-term cessation outcome data was not collected. Future research should replicate and extend this work, and investigate longitudinal relations of HL and cessation outcomes using longer follow-up periods. Studies should also investigate associations between HL and cessation outcomes using alternate measures of HL, such as the Chew HL items [36]. In addition, such associations should be examined among smokers not enrolled in cessation treatment. Finally, as previously noted, future studies should examine potential mechanisms underlying relations between low HL and poor cessation outcomes. Gaining a better understanding of the mechanisms through which HL influences cessation will help identify treatment targets in this population, improve current cessation interventions, and ultimately reduce tobacco-related health disparities and disease burden.

## Conclusions

This is the first study that we know of to investigate the association between HL and smoking cessation outcomes in a sample of low-SES, racially/ethnically diverse smokers enrolled in cessation treatment. Results indicated that lower HL smokers were more than three times as likely to relapse as smokers with higher HL, even after controlling for the effects of established predictors of relapse (e.g., education, income). Findings provide preliminary support for HL as an independent predictor of smoking relapse, and an important factor in understanding tobacco-related health disparities.

## Competing interests

The authors declare that they have no competing interests.

## Authors’ contributions

The data used in this manuscript are from a grant awarded to JIV. AJW, YL, and DWW were co-investigators on the grant and contributed heavily to the conceptualization and design of the overall project from which the data emanated. YL assisted with data analysis and interpretation. DWS drafted the manuscript and contributed to data analysis. All authors helped edit and refine the manuscript and assisted with conceptualization of the overall analytic plan. All authors read and approved the final manuscript.

## Pre-publication history

The pre-publication history for this paper can be accessed here:

http://www.biomedcentral.com/1471-2458/14/716/prepub
